# Transfusion of Blood Products and Clinical Outcomes for Patients With Dengue Fever: A Systematic Review and Meta-analysis

**DOI:** 10.1093/ofid/ofae507

**Published:** 2024-09-04

**Authors:** Zhi Jie Goh, Ruiqi Li, Min Xian Wang, Po Ying Chia, Jue Tao Lim

**Affiliations:** Lee Kong Chian School of Medicine, Nanyang Technological University, Singapore; Lee Kong Chian School of Medicine, Nanyang Technological University, Singapore; Centre for Population Health Research and Implementation, Singapore Health Services, Singapore; Lee Kong Chian School of Medicine, Nanyang Technological University, Singapore; Department of Infectious Diseases, National Centre for Infectious Diseases, Singapore; Department of Infectious Diseases, Tan Tock Seng Hospital, Singapore; Lee Kong Chian School of Medicine, Nanyang Technological University, Singapore

**Keywords:** blood product transfusion, dengue fever, length of hospital stay, mortality

## Abstract

**Background:**

This systematic review and meta-analysis aimed to analyze the effects of transfusing “nonpacked red blood cell” blood products in patients with dengue and evaluate the effectiveness in reducing mean hospital stay, bleeding, mortality rate, and intensive care unit requirements.

**Methods:**

Four databases were searched for relevant articles. Inclusion criteria were prospective or retrospective randomized or nonrandomized studies investigating the effects of transfusion of blood products in patients with dengue.

**Results:**

Nine studies were included in the final meta-analysis. Transfusion of blood products was associated with significantly higher mortality rate (9 studies; odds ratio [OR], 3.59 [95% confidence interval [CI], 1.07–15.98]; *I*^2^ = 0%; *P* = .04) and significantly longer mean hospital stay (6 studies; 0.56 day [95% CI, .03–1.08 day]; *I*^2^ = 95%; *P* = .04). There was no significant difference in the incidence of clinical bleeding (7 studies; OR, 1.13 [95% CI, .77–1.65]; *I*^2^ = 39%; *P* = .54) or intensive care unit requirement (3 studies; OR, 1.59 [.40–6.39]; *I*^2^ = 0%; *P* = .51).

**Conclusions:**

Transfusing blood products for patients with dengue showed no benefit and may even be harmful.

Dengue is a mosquito-borne viral disease with an estimated incidence rate of 105 million infections per year, of which 51 million infections result in febrile illness and 4 million symptomatic illnesses may require hospitalization [1]. In recent years, the incidence of dengue has increased greatly, and an increasing number of countries have reported local transmission of dengue [2]. Dengue outbreaks put a severe strain on the healthcare systems of affected countries, compromising their ability to provide care for patients [3]. Outbreaks of dengue have thus led to significant mortality rates and economic impacts in affected areas. These economic impacts arise not only from productivity losses among patients with dengue but also from unpaid informal caregivers, such as family members [4, 5].

Dengue presents clinically in 3 stages, the febrile, critical, and recovery stages. Acute high-grade fever is most often observed, though prolonged or saddleback fever patterns have also been reported [6]. The critical phase begins around defervescence and is associated with falling platelet counts and plasma leakage [7]. Warning signs of dengue include abdominal pain, mucosal bleeding, and plasma leakage, which may present in the critical phase. Patients with dengue who progress to severe dengue may experience shock, respiratory distress, severe bleeding, and severe organ dysfunction, resulting in death [8].

Current management for patients with dengue focused mainly on supportive care, such as fever and fluid management [9]. According to World Health Organization guidelines, red blood cell transfusions are recommended in cases with severe bleeding. While transfusions of other blood products, such as platelets and fresh frozen plasma, are practiced, there are concerns regarding their use as they may worsen fluid overload [10].

Various blood products have been proposed as management options for dengue fever. These include platelet transfusions, fresh frozen plasma, recombinant factor VIIa, intravenous immunoglobulin, and anti-D immunoglobulin. Platelet transfusion to correct thrombocytopenia has been proposed to be effective at reducing the severity of dengue infection [11]. Fresh frozen plasma is proposed to reduce immune-mediated destruction of platelets [12]. Thrombopoietin activator present in fresh frozen plasma may also stimulate thrombopoiesis [13].

To date, there have been no systematic reviews with meta-analysis regarding management practices in dengue, with prior reviews being qualitative [14]. The current review sought to address the apparent shortage in evidence-based guidelines for transfusion of “nonpacked red blood cell” blood products in dengue fever [15]. It also aimed to evaluate the role of nonpacked red blood cell blood product transfusion in the management of dengue and reduce the number of inappropriate transfusions with little evidence of benefit, which cause unnecessary complications.

## METHODS

### Search Strategy and Selection and Criteria

This study was reported in adherence to the Preferred Reporting Items for Systematic Reviews and Meta-analyses Statement (PRISMA) (Supplementary Data 1). Four databases (PubMed, Cochrane, Web of Science, and Cumulative Index to Nursing & Allied Health Literature) were searched for publications from inception of the databases through 10 August 2023. Citations of included articles were further searched manually for relevant articles.

The Medical Subject Headings (MeSH) terms and keywords searched included “dengue,” “dengue virus,” “blood product,” and “platelet transfusion.” This systematic review includes prospective or retrospective studies that are randomized or nonrandomized, which studied transfusion of blood products in patients with dengue and were published in English. Commentaries, studies that were conducted on nonhuman populations, or studies published in languages other than English were excluded. Further details on the search strategy used can be found in Supplementary Data 2. Each study was screened by 2 independent reviewers (Z. J. G. and R. L.), with conflicts resolved by a third independent reviewer (P. Y. C. or J. T. L.).

### Study Outcomes

The outcomes analyzed included death, length of hospital stay, intensive care unit requirement, and incidence of clinical bleeding. The definition of clinical bleeding excluded petechiae. Death, intensive care unit requirement, and incidence of clinical bleeding, as binary outcomes, were reported using odds ratios (ORs), while length of hospital stay, a continuous outcome, was reported using the pooled mean difference. Each outcome was reported with its respective 95% confidence interval (CI).

### Data Extraction and Assessment of Risk of Bias

Data extraction was conducted using a prespecified template (Supplementary Data 3). For randomized studies, the Cochrane Risk of Bias 2 (RoB 2) tool was used to evaluate risk of bias, while the Cochrane Risk of Bias in Non-randomised Studies—of Interventions (ROBINS-I) tool was used to evaluate the risk of bias for nonrandomized studies. Certainty of evidence was evaluated using the Grading of Recommendations, Assessment, Development, and Evaluations (GRADE) approach. In accordance with guidelines by Glisic et al [16], publication bias was evaluated through visual inspection of funnel plots.

### Analysis

Continuous data, reported in the forms of medians, ranges, or interquartile ranges, were converted to means and SDs using methods described by Wan et al [17], and the converted SDs were then aggregated for analysis. Statistical heterogeneity (inconsistency) was assessed as part of the GRADE approach, using *I*^2^ values. For outcomes with low heterogeneity, fixed-effects meta-analyses were conducted, while random-effects meta-analyses were conducted for outcomes with high heterogeneity. An *I*^2^ of ≤30% was considered low [18]. Data analysis was conducted using Cochrane Review Manager 5.4 software.

Subgroup analysis was conducted for outcomes with ≥5 studies included, and with ≥1 event reported in each subgroup. Subgroup analysis was conducted based on the type of blood product transfused (platelets or fresh frozen plasma), the age group of patients (pediatric or adults), or the type of study (randomized or nonrandomized study) investigated.

## RESULTS

### Study Details and Patient Demographics

**Figure 1.**
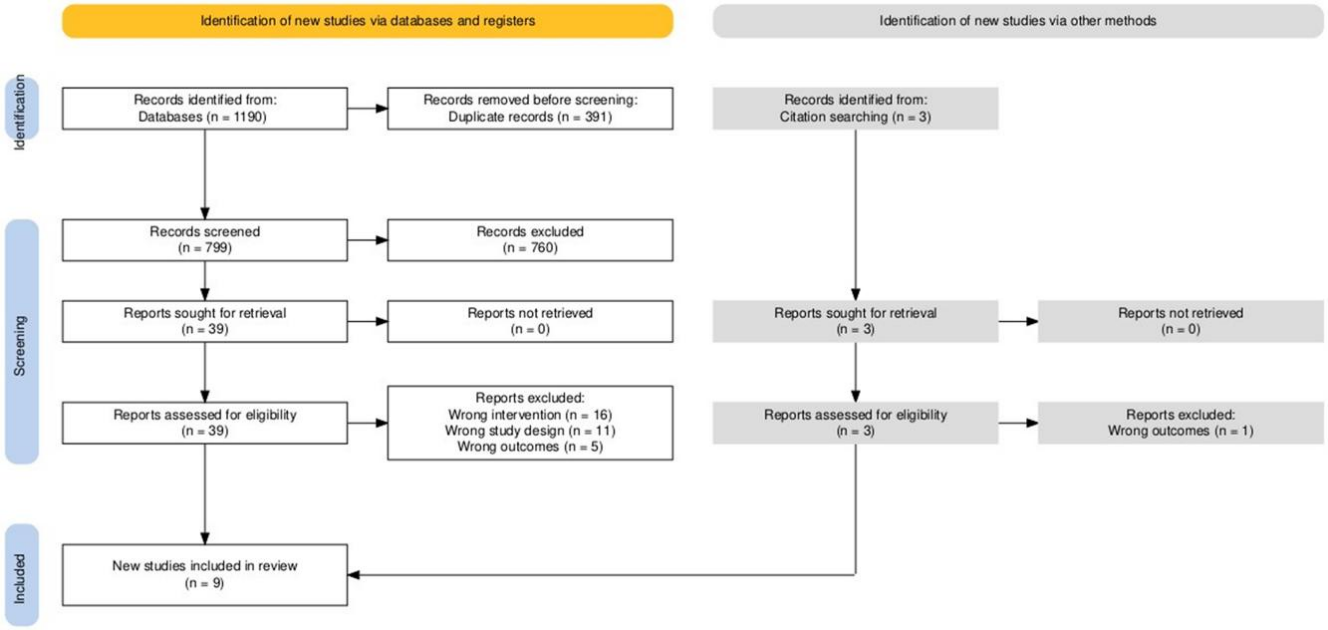
PRISMA (Preferred Reporting Items for Systematic Reviews and Meta-analyses Statement) flow diagram summarizing the numbers of databases searched, results obtained, and abstracts and full texts screened (diagram generated using programs designed by Haddaway et al [19]).

From 799 references, 43 studies were selected for full-text screening, and 9 were included in the meta-analysis (Figure 1) [12, 20–27]. In total, 1267 patients received transfusion, and 1174 patients did not. Two studies investigated pediatric patients, and 7 investigated adult patients. While not all studies reported data on the age of participants, the pooled mean ages of transfused (796 patients; mean [95% CI], 40.76 [40.02–41.50] years) and nontransfused (863 patients; 41.25 [40.55–41.95] years) adult patients were similar, as were the pooled mean ages of transfused (78 patients; 5.98 [5.36–6.59] years) and nontransfused (65 patients; 6.47 [5.78–7.15] years) pediatric patients. The proportions of male patients were also similar in the transfused (73.3%) and nontransfused (69.0%) groups. A majority of the nonrandomized studies made the decision to administer transfusion based on the clinical judgment of the physician. Six of the 9 studies investigated prophylactic transfusions, in patients who had no bleeding manifestations before the administration of transfusion. Further details of the patient demographics are summarized in Supplementary Data 4, further details of outcomes in Supplementary Data 5, and the types of blood products transfused in Table 1.

**Table 1.** Number of Studies by Type of Blood Product Transfused

Blood Product Transfused in Intervention Group	No. of Studies
Platelets only	7
Fresh frozen plasma only	1
Platelets and fresh frozen plasma	1

### Assessment of Study Quality

The risk of bias for the included studies are summarized in Supplementary Data 6 (for randomized studies) and Supplementary Data 7 (for nonrandomized studies). Two studies had an overall low risk of bias, 4 studies an overall moderate risk of bias, and 3 studies an overall high risk of bias. For nonrandomized studies, risks of bias mainly occurred due to significant confounding. The GRADE assessment of evidence is summarized in Supplementary Data 8. Visual inspection of the funnel plots did not reveal evidence of publication bias for any of the outcomes analyzed.

### Primary Meta-analysis

**Figure 2.**
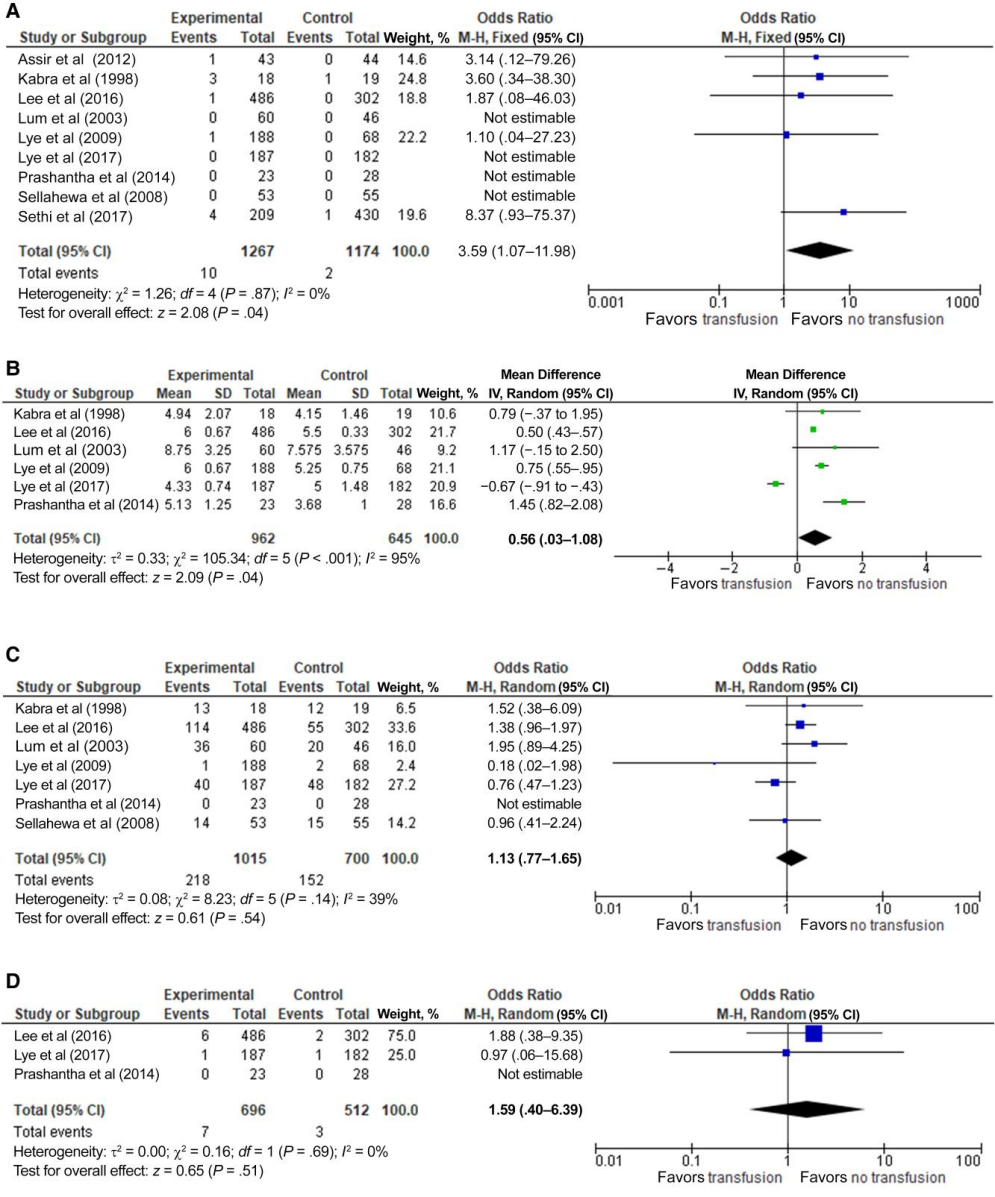
Forest plots for deaths (*A*), length of hospital stay (*B*), intensive care unit requirement (*C*), and incidence of clinical bleeding (*D*) [12, 20–27]. Abbreviations: CI, confidence interval; IV, Inverse variance; M-H, Mantel-Haenszel.

Transfusion of blood products was associated with significantly increased mortality rate (Figure 2*A*; 9 studies; OR, 3.59 [95% CI, 1.07–15.98]; *I*^2^ = 0%; *P* = .04). The mean hospital stays were significantly longer in the transfused than in the nontransfused group (Figure 2*B*; 6 studies; 0.56 day [95% CI, .03–1.08 day]; *I*^2^ = 95%; *P* = .04). Intensive care unit admissions did not differ significantly between both groups (Figure 2*C*; 3 studies; OR, 1.59 [95% CI, .40–6.39]; *I*^2^ = 0%; *P* = .51). The incidence of clinical bleeding did not differ significantly between groups (Figure 2*D*; 7 studies; OR, 1.13 [95% CI, .77–1.65]; *I*^2^ = 39%; *P* = .54).

### Subgroup Analysis

Forest plots for the various subgroup analysis below are detailed in Supplementary Data 9. Comparing studies that transfused platelets with those that transfused fresh frozen plasma, no significant subgroup effect was observed for the length of hospital stay (*P* = .35) or the incidence of bleeding (*P* = .47). Comparing studies that transfused pediatric patients with those that transfused adult patients, no significant subgroup effect was observed for deaths (*P* > .99), length of hospital stay (*P* = .35), or incidence of bleeding (*P* = .13).

Comparing randomized controlled trials with nonrandomized trials, no significant subgroup effect was observed for the outcomes of death (*P* = .93) or bleeding (*P* = .06). While randomized controlled trials may appear to have a shorter hospital stays with transfusion of blood products, this is a limitation of the statistical methods used in estimating means and SDs, and the study analyzed reported no significant difference in the length of hospital stay between transfused and nontransfused groups.

### Other Findings

A death related to transfusion was noted in one of the trials [20], in which 1 patient succumbed to transfusion-related acute lung injury directly related to platelet transfusion. Moreover, other studies also reported adverse reactions to transfusion, such as fever, itching, and anaphylaxis, though no further deaths were attributed to transfusion [22, 27].

## DISCUSSION

Severe bleeding, a symptom of severe dengue, is responsible for a significant number of deaths [28]. Literature sources disagree on whether platelet counts were correlated with bleeding manifestations in patients with dengue. However, current evidence suggests that there is no correlation between the extent of thrombocytopenia and the incidence of severe bleeding in dengue [29]. The strongest risk factors for bleeding in patients with dengue include shock of extended duration, as well as low hematocrit [30]. These cannot be corrected by transfusion of platelets, fresh frozen plasma, or immunoglobulins.

Paradoxically, the use of platelet transfusion was not associated with an increase in the rate of recovery of platelet counts. In fact, several studies reported unexpected slower recovery of platelet counts in transfused patients, compared with nontransfused patients [24, 27]. Another randomized study reported no significant difference in the recovery of platelet counts between transfused and nontransfused groups [22]. It was postulated that this may be due to a decrease in thrombopoietin level in transfused patients, reducing the rate of endogenous platelet production [31]. Conversely, patients who received fresh frozen plasma had a faster recovery of platelet counts, compared with the control group [12].

According to the 2009 World Health Organization guidelines, patients with dengue may be discharged from the hospital only if they had an increasing trend of platelet counts and have stable hematocrits without intravenous fluid transfusion [10]. In addition, some of the studies analyzed did not discharge patients until their platelet counts exceeded 50 000/µL, following the recommendation in the national guidelines for India [24]. The delay in platelet count increase in patients who received platelet transfusions, who comprised a majority of patients in this meta-analysis, could have contributed to the observed trend. The studies that investigated transfusions of anti-D immunoglobulin or recombinant factor VIIa also did not report significant differences in the length of hospital stay in transfused patients compared with the control group.

Increased deaths and intensive care admissions in patients receiving transfusions could also have been attributed to adverse events occurring as a direct consequence of transfusion. The studies analyzed reported patient deaths that were directly correlated to transfusion, including deaths from transfusion-associated acute lung injury [20]. Other adverse events that occurred in higher frequencies in the transfused groups, compared with control groups, included anaphylaxis and fluid overload.

To date, the current study is the first systematic review with meta-analysis conducted on transfusion practices for patients with dengue. IT analyzed results from 9 prior studies, which included a total of 2441 patients. Furthermore, the GRADE approach was used to evaluate the certainty of evidence, which allowed a more holistic and clear presentation of the results obtained.

However, this study still has several limitations. The inclusion of nonrandomized trials in this meta-analysis could have introduced selection bias and unobserved confounding into the analysis, as some trials reported that patients in the transfused group were initially more clinically unwell. This was addressed partially by subgroup analysis or propensity score matching procedures in some of the studies [24, 26, 27]. Furthermore, there was also variability in patient demographics among the studies analyzed, with some studies including pediatric patients alone. These limitations could be addressed with better-designed randomized controlled trials evaluating the role of transfusion of different blood products in the management of dengue.

The validity of the results obtained could have been limited by the inclusion of heterogenous study designs, such as both randomized and nonrandomized trials. Many of the nonrandomized trials were potentially affected by selection bias for treatment. In these trials, it was often noted that a larger proportion of the patients with dengue who received transfusions had more complicated dengue, due to the different management practices or transfusion guidelines of the hospitals where the trials were conducted [24, 27, 32]. During evaluation of risk of bias, nonrandomized trials with significant differences in the proportion of patients with warning signs or severe dengue between the transfused and nontransfused groups were all assigned a high risk of bias due to serious confounding. Comparing subgroups of randomized controlled trials against nonrandomized studies, nonrandomized studies reported significant increases in the duration of hospital stay, but randomized studies did not. This suggests that the results obtained for this outcome could have been affected by selection bias.

Furthermore, the classification of clinical bleeding was limited by the varied definitions of bleeding adopted by the various studies. While it was not possible to determine the severity of bleeding for every study, a consensus among the included studies was to exclude petechial bleeding from the definition of clinical bleeding. However, this may be less effective at representing the bleeding risk to patients. In one study, while only a single patient in the transfusion group experienced bleeding, that patient had severe gastrointestinal bleeding and died. Conversely, the 2 patients in the control group with bleeding had only mild bleeding, which required no further intervention [24].

Statistical methods used could have contributed to the inaccuracy of results, such as the estimations used in converting medians, interquartile ranges, and ranges to means and SDs. Some studies reported results using 5th–95th percentile ranges, which were approximated as 0th–100th percentile ranges in data analysis. Furthermore, the length of hospital stay was reported to only 1 significant figure in some studies, which affected the accuracy of the estimations used, as some comparisons that were reported as not significant in the original study became significant after conversion [22].

The majority of studies involved transfusion of platelets, while only 2 of 9 studies, involving 214 of 2441 patients, studied the transfusion of blood products other than platelets. Thus, there is a need for high-quality randomized controlled trials investigating the transfusion of these other blood products, such as fresh frozen plasma

In conclusion, the results of this study suggest that current transfusion practices with nonpacked red blood cell blood products in patients with dengue are not beneficial and potentially harmful. These were corroborated with previous literature reporting potential harms of platelet transfusion as well as lack of evidence of benefit with transfusion of other types of blood products, such as fresh frozen plasma. Further research into transfusion of nonpacked red blood cell blood products other than platelets is needed, to enable clinicians to make better decisions in the management of patients with dengue.

## Supplementary Material

ofae507_Supplementary_Data

## Supplementary Data

Supplementary materials are available at *Open Forum Infectious Diseases* online. Consisting of data provided by the authors to benefit the reader, the posted materials are not copyedited and are the sole responsibility of the authors, so questions or comments should be addressed to the corresponding author.
