# Supplementary material for: Transfusion of Blood Products and Clinical Outcomes for Patients With Dengue Fever: A Systematic Review and Meta-analysis
Source: Open Forum Infect Dis. 2024 Sep 4;11(9):ofae507. doi: 10.1093/ofid/ofae507 (PMC11420673; doi:10.1093/ofid/ofae507)
Supplement: ofae507_Supplementary_Data [file ofae507_supplementary_data.docx]

Table S1. Preferred Reporting Items for Systematic Reviews and Meta-analyses

Table S2. Search Strategy

Table S3. Data Extraction Template

Table S4. Demographics of Included Studies

Table S5. Outcomes

Table S6. Cochrane Risk of Bias Tool 2

Table S7. Cochrane Risk of Bias in Non-randomised Studies - of Interventions assessment tool

Table S8. Grading of Recommendations, Assessment, Development, and Evaluations

Table S9. Subgroup Analysis

Table S1. Preferred Reporting Items for Systematic Reviews and Meta-analyses

| Section and  Topic | Item  # | Checklist item | Location  where item is reported |
| --- | --- | --- | --- |
| Title | | |  |
| Title | 1 | Identify the report as a systematic review. | 1 |
| Abstract | | |  |
| Abstract | 2 | See the PRISMA 2020 for Abstracts checklist. | 2 |
| Introduction | | |  |
| Rationale | 3 | Describe the rationale for the review in the context of existing knowledge. | 4 |
| Objectives | 4 | Provide an explicit statement of the objective(s) or question(s) the review addresses. | 5 |
| Methods | | |  |
| Eligibility criteria | 5 | Specify the inclusion and exclusion criteria for the review and how studies were grouped for the syntheses. | 5-6 |
| Information  sources | 6 | Specify all databases, registers, websites, organisations, reference lists and other sources searched or consulted to identify studies. Specify the date when each source was last searched or consulted. | 5 |
| Search strategy | 7 | Present the full search strategies for all databases, registers and websites, including any filters and limits used. | Supplementary Data S2 |
| Selection process | 8 | Specify the methods used to decide whether a study met the inclusion criteria of the review, including how many reviewers screened each record and each report retrieved, whether they worked independently, and if applicable, details of automation tools used in the process. | 6 |
| Data collection  process | 9 | Specify the methods used to collect data from reports, including how many reviewers collected data from each report, whether they worked independently, any processes for obtaining or confirming data from study investigators, and if applicable, details of automation tools used in the process. | 7 |
| Data items | 10a | List and define all outcomes for which data were sought. Specify whether all results that were compatible with each outcome domain in each study were sought (e.g. for all measures, time points, analyses), and if not, the methods used to decide which results to collect. | 7 |
|  | 10b | List and define all other variables for which data were sought (e.g. participant and intervention characteristics, funding sources). Describe any assumptions made about any missing or unclear information. | Supplementary Data S4 |
| Study risk of bias assessment | 11 | Specify the methods used to assess risk of bias in the included studies, including details of the tool(s) used, how many reviewers assessed each study and whether they worked independently, and if applicable, details of automation tools used in the process. | 7 |
| Effect measures | 12 | Specify for each outcome the effect measure(s) (e.g. risk ratio, mean difference) used in the synthesis or presentation of results. | 7 |
| Synthesis  methods | 13a | Describe the processes used to decide which studies were eligible for each synthesis (e.g. tabulating the study intervention characteristics and comparing against the planned groups for each synthesis (item #5)). | 7 |
|  | 13b | Describe any methods required to prepare the data for presentation or synthesis, such as handling of missing summary statistics, or data conversions. | 7 |
|  | 13c | Describe any methods used to tabulate or visually display results of individual studies and syntheses. | 7 |
|  | 13d | Describe any methods used to synthesize results and provide a rationale for the choice(s). If meta-analysis was performed, describe the model(s), method(s) to identify the presence and extent of statistical heterogeneity, and software package(s) used. | 7 |
|  | 13e | Describe any methods used to explore possible causes of heterogeneity among study results (e.g. subgroup analysis, meta-regression). | 7 |
|  | 13f | Describe any sensitivity analyses conducted to assess robustness of the synthesized results. | 7 |
| Reporting bias  assessment | 14 | Describe any methods used to assess risk of bias due to missing results in a synthesis (arising from reporting biases). | 7 |
| Certainty  assessment | 15 | Describe any methods used to assess certainty (or confidence) in the body of evidence for an outcome. | 7 |

**PRISMA 2020 Checklist**

| Section and  Topic | Item  # | Checklist item | Location  where item is reported |
| --- | --- | --- | --- |
| Results | | |  |
| Study selection | 16a | Describe the results of the search and selection process, from the number of records identified in the search to the number of studies included in the review, ideally using a flow diagram. | 8 |
|  | 16b | Cite studies that might appear to meet the inclusion criteria, but which were excluded, and explain why they were excluded. | NA |
| Study  characteristics | 17 | Cite each included study and present its characteristics. | 8 |
| Risk of bias in  studies | 18 | Present assessments of risk of bias for each included study. | Supplementary Data S6 and S7 |
| Results of  individual studies | 19 | For all outcomes, present, for each study: (a) summary statistics for each group (where appropriate) and (b) an effect estimate and its precision (e.g. confidence/credible interval), ideally using structured tables or plots. | 7 |
| Results of  syntheses | 20a | For each synthesis, briefly summarise the characteristics and risk of bias among contributing studies. | 9 |
|  | 20b | Present results of all statistical syntheses conducted. If meta-analysis was done, present for each the summary estimate and its precision (e.g. confidence/credible interval) and measures of statistical heterogeneity. If comparing groups, describe the direction of the effect. | 9-10 |
|  | 20c | Present results of all investigations of possible causes of heterogeneity among study results. | 11-12 |
|  | 20d | Present results of all sensitivity analyses conducted to assess the robustness of the synthesized results. | 11-12 |
| Reporting biases | 21 | Present assessments of risk of bias due to missing results (arising from reporting biases) for each synthesis assessed. | NA |
| Certainty of  evidence | 22 | Present assessments of certainty (or confidence) in the body of evidence for each outcome assessed. | Supplementary Data S8 |
| Discussion | | |  |
| Discussion | 23a | Provide a general interpretation of the results in the context of other evidence. | 12 |
|  | 23b | Discuss any limitations of the evidence included in the review. | 14 |
|  | 23c | Discuss any limitations of the review processes used. | 14 |
|  | 23d | Discuss implications of the results for practice, policy, and future research. | 14 |
| Other information | | |  |
| Registration and protocol | 24a | Provide registration information for the review, including register name and registration number, or state that the review was not registered. | NA |
|  | 24b | Indicate where the review protocol can be accessed, or state that a protocol was not prepared. | NA |
|  | 24c | Describe and explain any amendments to information provided at registration or in the protocol. | NA |
| Support | 25 | Describe sources of financial or non-financial support for the review, and the role of the funders or sponsors in the review. | NA |
| Competing  interests | 26 | Declare any competing interests of review authors. | NA |
| Availability of  data, code and  other materials | 27 | Report which of the following are publicly available and where they can be found: template data collection forms; data extracted from included studies; data used for all analyses; analytic code; any other materials used in the review. | Supplementary Data S3; S4-5; NA; NA |

**Abbreviations:** NA: not applicable

Table S2. Search Strategy

**PubMed**

| Number | Query | Results |
| --- | --- | --- |
| 1 | Dengue [MeSH] OR Dengue Virus [MeSH] | 19367 |
| 2 | “Dengue” | 29375 |
| 3 | Blood transfusion [MeSH] OR Platelet Transfusion [MeSH] OR Fibrinogen [MeSH] OR Fresh Frozen Plasma [MeSH] | 161754 |
| 4 | “Blood transfusion” OR “Blood product” OR “Platelet transfusion” OR “whole blood” OR “Fibrinogen” OR “FFP” OR “fresh frozen plasma” OR “cryoprecipitate” | 240892 |
| 5 | (#1 OR #2) AND (#3 OR #4) | **506** |

**Cochrane**

| Number | Query | Results |
| --- | --- | --- |
| 1 | MeSH descriptor: [Dengue] explode all trees | 434 |
| 2 | MeSH descriptor: [Dengue Virus] explode all trees | 143 |
| 3 | “Dengue” | 901 |
| 4 | MeSH descriptor: [Blood Transfusion] explode all trees | 4927 |
| 5 | MeSH descriptor: [Platelet Transfusion] explode all trees | 375 |
| 6 | MeSH descriptor: [Fibrinogen] explode all trees | 1872 |
| 7 | “Blood transfu*” OR “Blood product” OR “Platelet transfu*” OR “whole blood” OR “Fibrinogen” OR “FFP” OR “fresh frozen plasma” OR “cryoprecipitate” | 12816 |
| 8 | (#1 OR #2 OR #3) AND (#4 OR #5 OR #6 OR #7) | **30** |

**CINAHL**

| Number | Query | Results |
| --- | --- | --- |
| 1 | (MH "Dengue") | 2766 |
| 2 | “Dengue” | 4284 |
| 3 | (MH “Blood Transfusion”) | 14284 |
| 4 | (MH “Platelet Transfusion”) | 1765 |
| 5 | (MH “Fibrinogen”) | 3040 |
| 6 | “Blood transfu*” OR “Blood product” OR “Platelet transfu*” OR “whole blood” OR “full blood” OR “Fibrinogen” OR “FFP” OR “fresh frozen plasma” OR “cryoprecipitate” | 39037 |
| 7 | S1 OR S2 | 4284 |
| 8 | S3 OR S4 OR S5 OR S6 | 39037 |
| 9 | S7 AND S8 | **125** |

**Web of Science**

| Number | Query | Results |
| --- | --- | --- |
| 1 | TS=(“Dengue”) | 38119 |
| 2 | TS=(“Dengue Virus”) | 16262 |
| 3 | TS=(“Blood Transfusion”) | 50375 |
| 4 | TS=("Platelet Transfusion") | 6754 |
| 5 | TS=(“Fresh Frozen Plasma”) | 7827 |
| 6 | TS=("Fibrinogen") | 55381 |
| 7 | TS=(“Blood transfu*” OR “Blood product” OR “Platelet transfu*” OR “whole blood” OR “Fibrinogen” OR “FFP” OR “fresh frozen plasma” OR “cryoprecipitate”) | 189702 |
| 8 | #1 OR #2 | 4284 |
| 9 | #3 OR #4 OR #5 OR #6 OR #7 | 39037 |
| 10 | #8 AND #9 | **540** |

Table S3. Data Extraction Template

**Study Details**

| Study ID | E.g. Smith 2021 |
| --- | --- |
| Title |  |
| Journal |  |
| Country |  |
| Hospital |  |
| Inclusion Criteria |  |
| Exclusion Criteria |  |
| Start Date |  |
| End Date |  |
| Interventions in Experimental Group |  |
| Interventions in Control Group |  |

**Patient Demographics**

|  | Transfused Group | Control Group |
| --- | --- | --- |
| Sample Size |  |  |
| Male Patients |  |  |
| Age |  |  |
| Dengue Severity Classification |  |  |
| Comorbidities |  |  |
| Others |  |  |

**Outcomes**

|  | Transfused Group | Control Group |
| --- | --- | --- |
| Mortality |  |  |
| Length of Hospital Stay |  |  |
| Intensive Care Unit Requirement |  |  |
| Bleeding |  |  |

**Risk of Bias Assessment - Cochrane Risk of Bias 2**

| Item | Verdict | Evidence |
| --- | --- | --- |
| Randomisation |  |  |
| Deviations |  |  |
| Missing Outcomes |  |  |
| Measurement |  |  |
| Selection |  |  |

**Risk of Bias Assessment - Cochrane Risk of Bias in Non-Randomised Studies - of Interventions**

| Item | Verdict | Evidence |
| --- | --- | --- |
| Confounding |  |  |
| Selection |  |  |
| Classification |  |  |
| Deviations |  |  |
| Missing data |  |  |
| Measurements |  |  |
| Selection of Result |  |  |

Table S4. Demographics of Included Studies

| Study ID | Country | Sample size | Age | Males | Dengue Severity | Indication for  Transfusion |
| --- | --- | --- | --- | --- | --- | --- |
| Assir 2012 | Pakistan | TG: 43  CG: 44 | TG: 33(15-65)  CG: 36(16-78) | NR | TG: DF 17, DHF1 8, DHF2 18  CG: DF 20, DHF1 6, DHF2 18 | Randomised |
| Kabra 1998 | India | TG: 18  CG: 19 | TG: 6.65±2.87  CG: 7.41±2.32 | TG: 11  CG: 9 | TG: Dengue severity classification: 4 II, 9 III, 4 IV  CG: Dengue severity classification: 5 II, 8 III, 4 IV | Clinical judgement of  the attending  physician |
| Lee 2016 | Singapore | TG: 486  CG: 302 | TG: 40(22-65)*  CG: 40(21-67)* | TG: 356  CG: 219 | TG: Probable dengue 301, DHF 44, DSS 4, severe 18  CG: Probable dengue 485, DHF 83, DSS 7, severe 68 | Clinical judgement of  the in-charge  physician |
| LumLCS 2003 | Malaysia | TG: 60  CG: 46 | TG: 6(0.1-11.0)*  CG: 6(0.3-12.0)* | TG: 33  CG: 27 | NR | Clinical judgement of  the attending  physician and the  prevailing transfusion  practice |
| Lye 2009 | Singapore | TG: 188  CG: 68 | TG: 40(22-64)*  CG: 38(22-58)* | TG: 144  CG: 45 | TG: DHF 4  CG: DHF 2 | Clinical judgement of  the physician |
| Lye 2017 | Singapore, Malaysia | TG: 187  CG: 182 | TG: 44.3±14.1  CG: 45.2±12.4 | TG: 139  CG: 140 | TG: 32 diagnosed PCR positive  CG: 28 diagnosed PCR positive | Randomised |
| Prashantha 2014 | India | TG: 23  CG: 28 | TG: mean 29, range 18-38  CG: mean 28.5, range 18-44 | TG: 19  CG: 17 | NR | Religious belief of the patient |
| Sellahewa 2008 | Sri Lanka | TG: 53  CG: 55 | TG: 28.8±11.8  CG: 30±11.1 | TG: 42  CG: 39 | NR | Randomised |
| Sethi 2017 | Pakistan | TG: 209  CG: 430 | NR | TG: 155  CG: 274 | NR | NR |

**Abbreviations:** CG: control group; DHF: dengue haemorrhagic fever; DF: dengue fever; DSS: dengue shock syndrome; NR: not reported; PCR: polymerase chain reaction; TG: transfused group

For data on age of subjects, data are presented as mean ± standard deviation, median(range), or median(5th to 95th percentile range)* unless otherwise stated.

Table S5. Outcomes

| Study ID | Mortality | Length of Hospital Stay (days) | Intensive Care Unit Requirement | Bleeding |
| --- | --- | --- | --- | --- |
| Assir 2012 | TG: 1  CG: 0 | NR | NR | NR |
| Kabra 1998 | TG: 3  CG: 1 | TG: 4.94±2.07  CG: 4.15±1.46 | TG: 1  CG: 1 | TG: 13  CG: 12 |
| Lee 2016 | TG: 1  CG: 0 | TG: 6(4-8)  CG: 5(5-7) | NR | TG: 114  CG: 55 |
| LumLCS 2003 | TG: 0  CG: 0 | TG: 7(4.0-17.0)  CG: 5(3.0-17.3) | NR | TG: 36  CG: 20 |
| Lye 2009 | TG: 1  CG: 0 | TG: 6(4-8)  CG: 5(4-7) | NR | TG: 1  CG: 2 |
| Lye 2017 | TG: 0  CG: 0 | TG: median 4, IQR 4-5  CG: median 5, IQR 4-6 | TG: 1  CG: 1 | TG: 40  CG: 48 |
| Prashantha 2014 | TG: 0  CG: 0 | TG: mean 5.13, range 4-9  CG: mean 3.68, range 2-6 | TG: 0 CG: 0 | NR |
| Sellahewa 2008 | TG: 0  CG: 0 | NR | NR | TG: 14  CG: 15 |
| Sethi 2017 | TG: 4  CG: 1 | NR | NR | NR |

**Abbreviations:** CG: control group; IQR: interquartile range; NR: not reported; TG: transfused group;

For length of hospital stay, data are presented as mean ± standard deviation, or median (5th to 95th percentile range), unless otherwise stated

Table S6. Cochrane Risk of Bias Tool 2

**
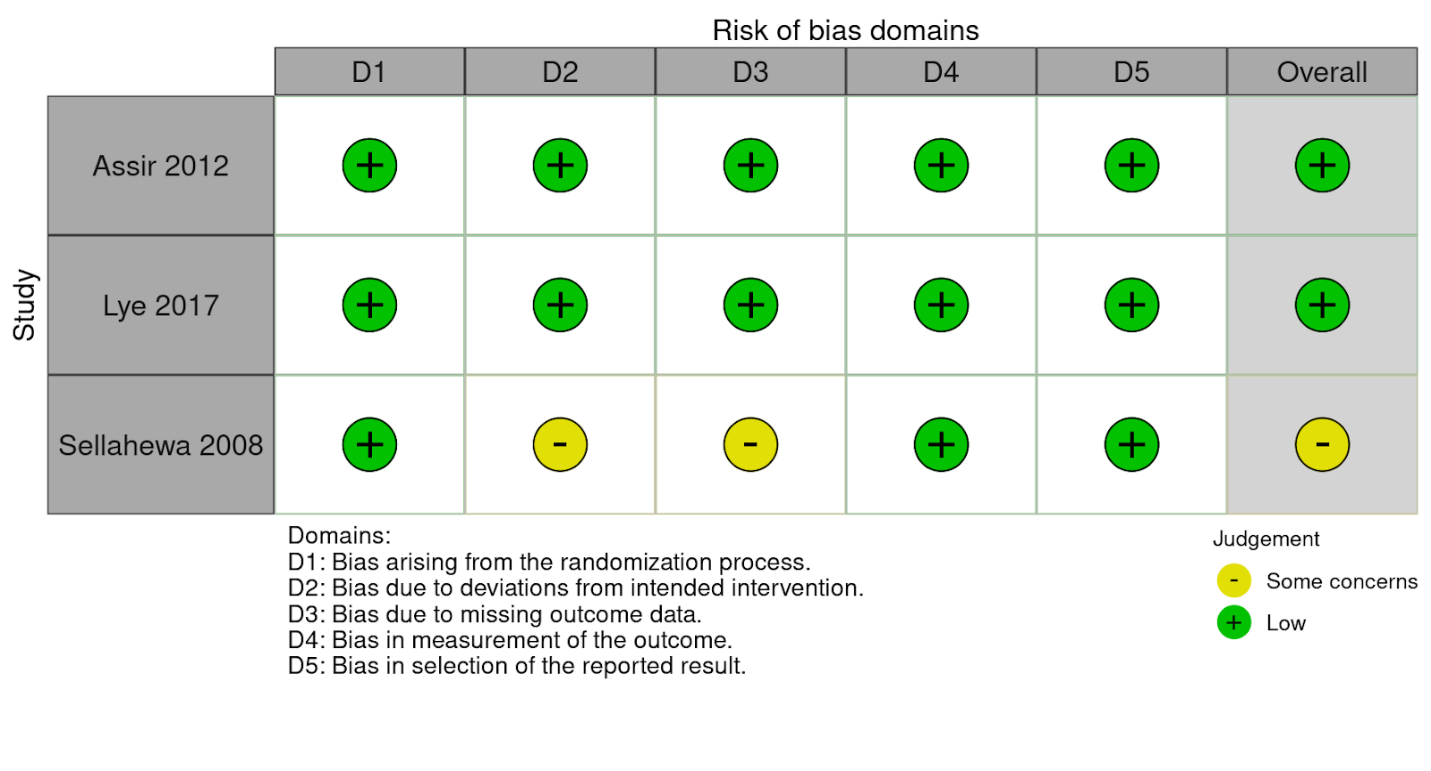
**

*
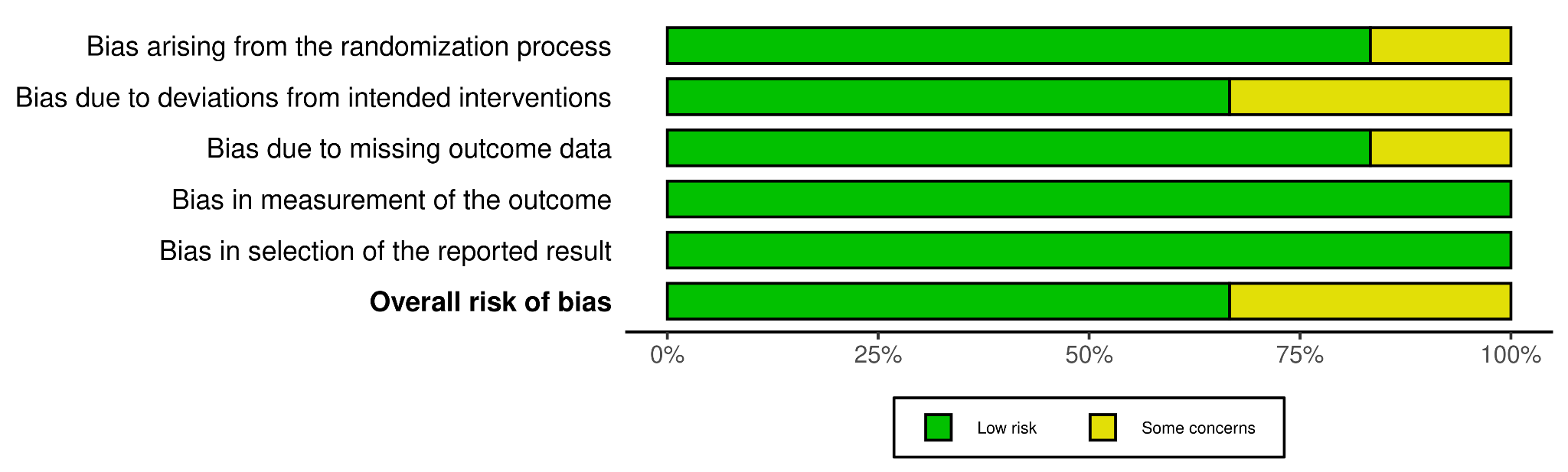
*

Table S7. Cochrane Risk of Bias in Non-randomised Studies - of Interventions assessment tool

**
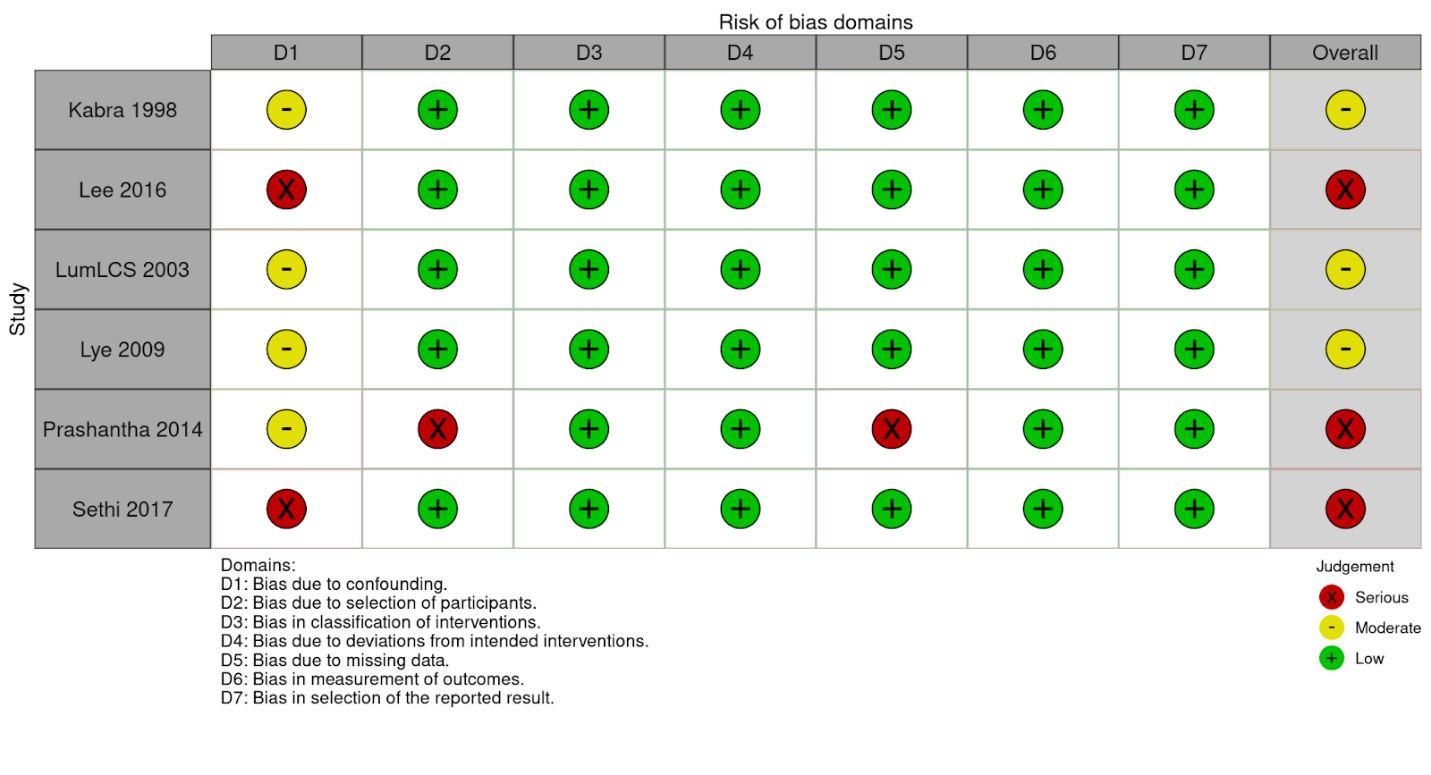
**

**
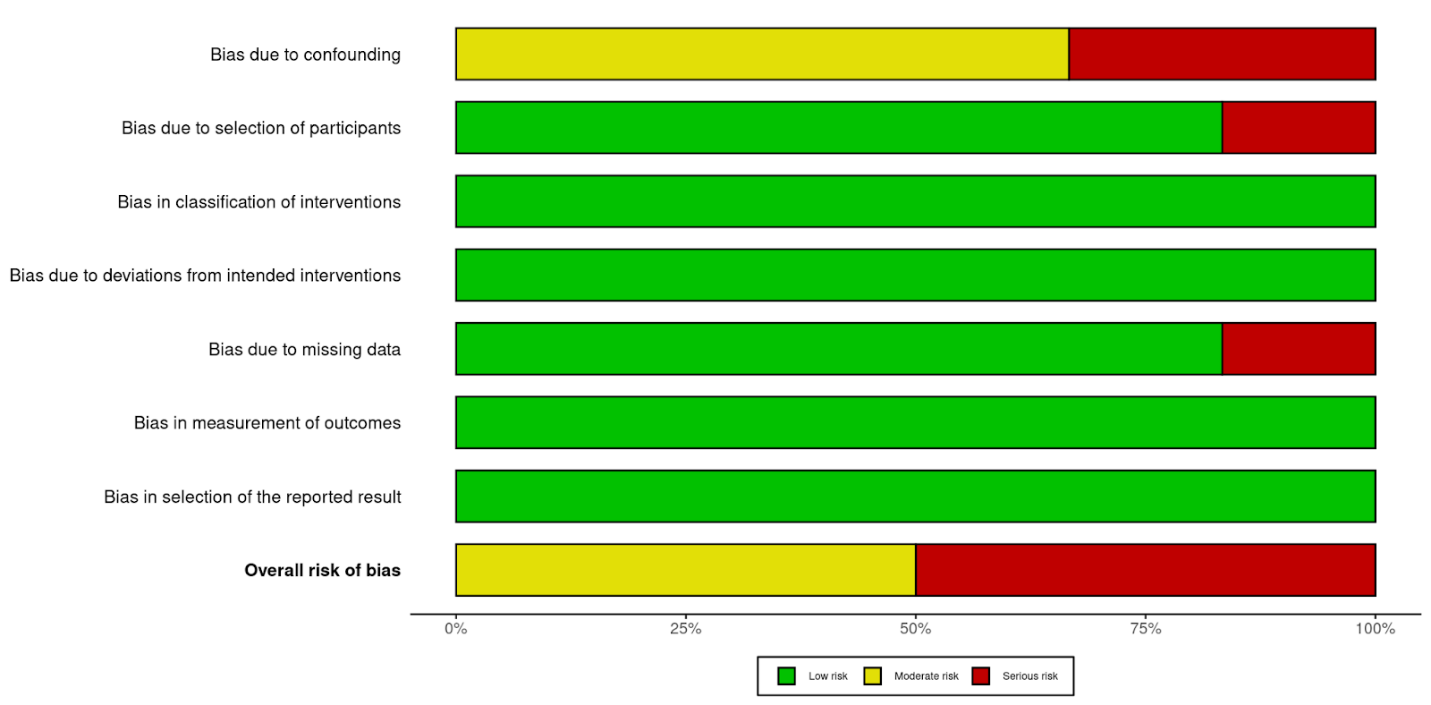
**

Table S8. Grading of Recommendations, Assessment, Development, and Evaluations (GRADE)

**GRADE for all studies**

| Outcome | Number of participants (Studies) | Relative effect (95%-CI) | Illustrative comparative risk - with transfusion | Illustrative comparative risk - without transfusion | Certainty of the evidence (GRADE) |
| --- | --- | --- | --- | --- | --- |
| Mortality | 2441 (9) | OR 3.59 (1.07–11.98) | 1 in 125 patients | 1 in 500 patients | ⊕⊕⊕◯  MODERATE due to risk of bias |
| Length of hospital stay | 1607 (6) | MD 0.56 days (0.03–1.08 days) | 5.81 days | 5.25 days (0.56 days shorter) | ⊕◯◯◯  VERY LOW  due to risk of bias and inconsistency |
| Incidence of clinical bleeding | 1715 (7) | OR 1.13 (0.77–1.65) | 1 in 5 patients | 1 in 5 patients | ⊕⊕◯◯  LOW  due to risk of bias and imprecision |
| Intensive care unit requirement | 1208 (3) | OR 1.59 (0.40–6.39) | 1 in 100 patients | 1 in 170 patients | ⊕◯◯◯  VERY LOW  due to risk of bias and imprecision |

**GRADE for randomised studies only**

| Outcome | Number of participants (Studies) | Relative effect (95%-CI) | Illustrative comparative risk - with transfusion | Illustrative comparative risk - without transfusion | Certainty of the evidence (GRADE) |
| --- | --- | --- | --- | --- | --- |
| Mortality | 564 (3) | OR 3.12 (0.12–79.26) | 1 in 300 patients | 1 in 1000 patients | ⊕⊕⊕◯  MODERATE due to imprecision |
| Incidence of clinical bleeding | 477 (2) | OR 0.80 (0.53–1.22) | 1 in 5 patients | 1 in 4 patients | ⊕⊕⊕◯  MODERATE due to imprecision |

**Abbreviations:** CI: confidence interval, MD: mean difference, OR: odds ratio

Table S9. Subgroup Analysis

**Analysed by type of blood product transfuse**

Mortality

No subgroup analysis was conducted as no mortality occurred in either the transfusion or control groups of both studies.

Length of hospital stay

**
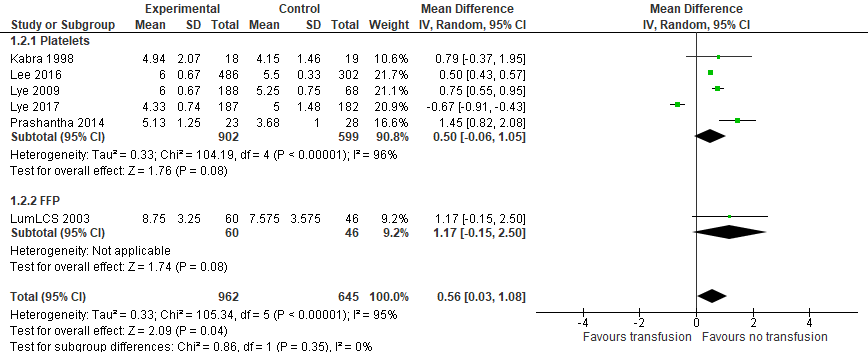
**

Bleeding

**
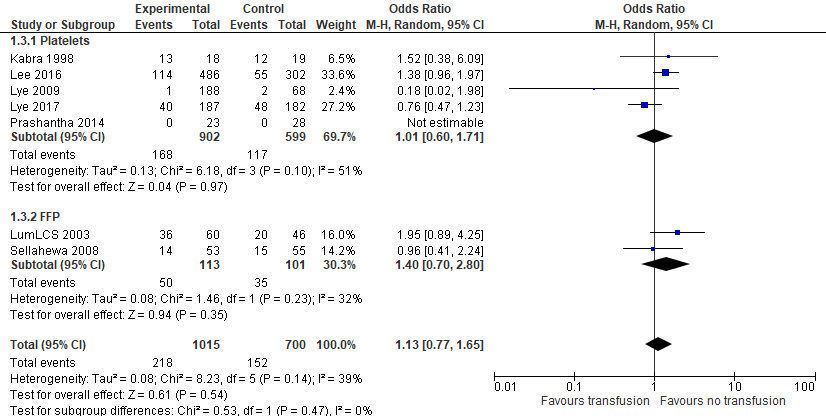
**

**Analysed by age of patients (Adult vs paediatric)**

Mortality

**
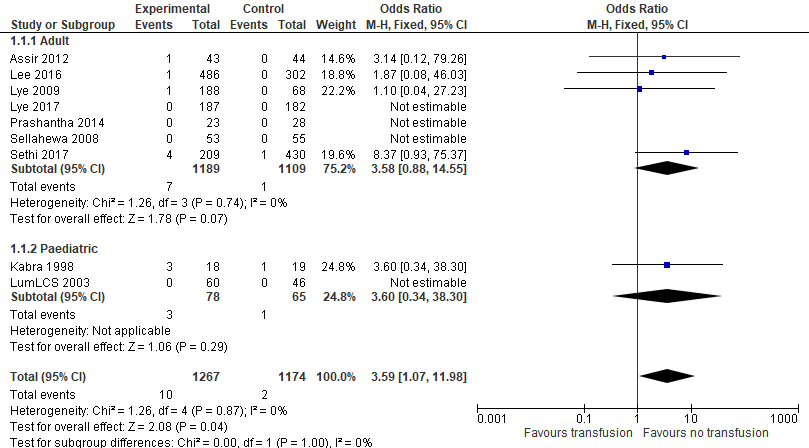
**

Length of hospital stay

**
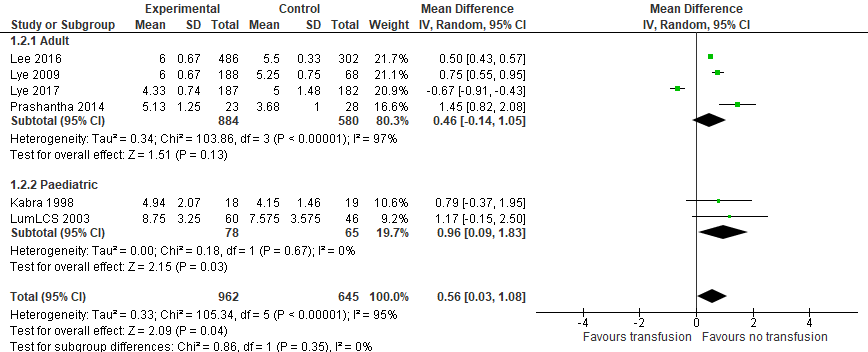
**

Bleeding

**
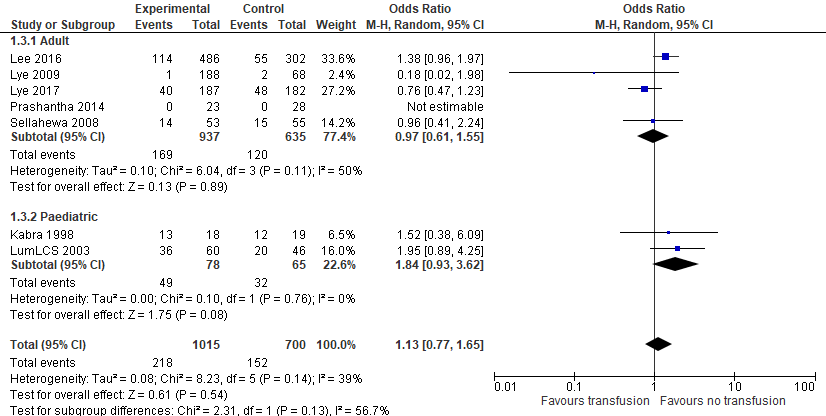
**

**Analysed by type of study (Randomised vs non-randomised)**

Mortality

**
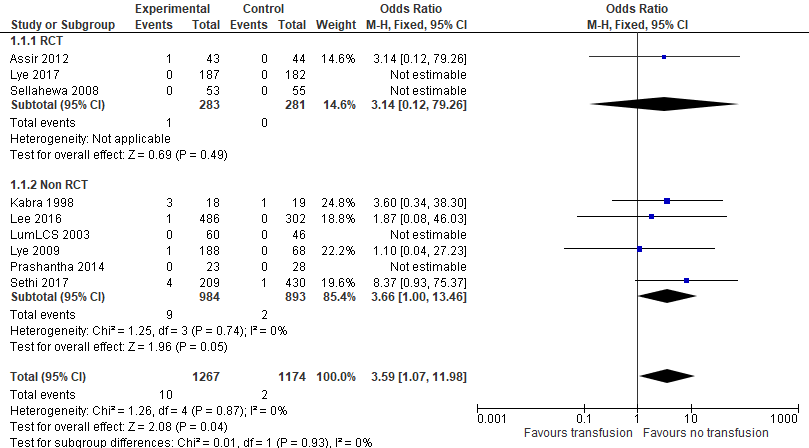
**

Length of hospital stay

**
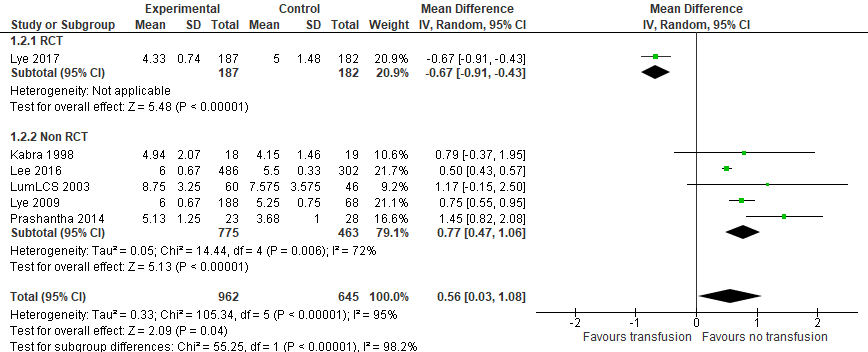
**

Bleeding

**
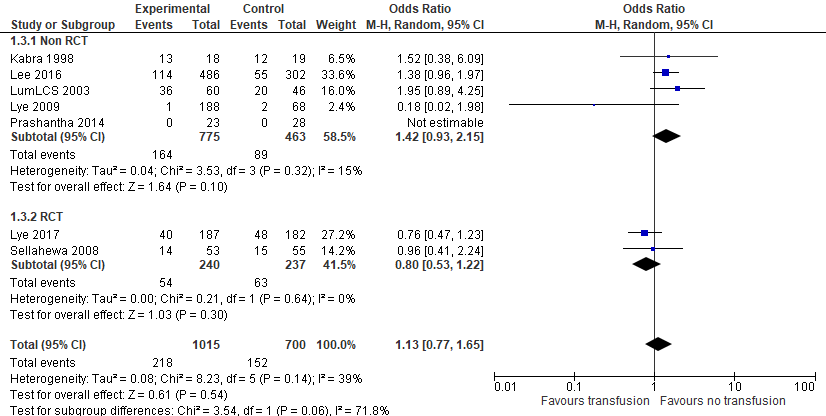
**
